# The Relationship Among Intestinal Bacteria, Vitamin K and Response of Vitamin K Antagonist: A Review of Evidence and Potential Mechanism

**DOI:** 10.3389/fmed.2022.829304

**Published:** 2022-04-18

**Authors:** Han Yan, Yi Chen, Hong Zhu, Wei-Hua Huang, Xin-He Cai, Dan Li, Ya-Juan Lv, Si-Zhao, Hong-Hao Zhou, Fan-Yan Luo, Wei Zhang, Xi Li

**Affiliations:** ^1^Department of Pharmacy, The Second Xiangya Hospital, Central South University, Changsha, China; ^2^Department of Clinical Pharmacology, Xiangya Hospital, Central South University, Changsha, China; ^3^Institute of Clinical Pharmacology, Central South University, Hunan Key Laboratory of Pharmacogenetics, Changsha, China; ^4^National Clinical Research Center for Geriatric Disorders, Xiangya Hospital, Central South University, Changsha, China; ^5^Department of Cardiovascular Surgery, Xiangya Hospital, Central South University, Changsha, China

**Keywords:** vitamin K, intestinal bacteria, butyrate, vitamin K antagonist, lithocholic acid

## Abstract

The vitamin K antagonist is a commonly prescribed effective oral anticoagulant with a narrow therapeutic range, and the dose requirements for different patients varied greatly. In recent years, studies on human intestinal microbiome have provided many valuable insights into disease development and drug reactions. A lot of studies indicated the potential relationship between microbiome and the vitamin K antagonist. Vitamin K is absorbed by the gut, and the intestinal bacteria are a major source of vitamin K in human body. A combined use of the vitamin K antagonist and antibiotics may result in an increase in INR, thus elevating the risk of bleeding, while vitamin K supplementation can improve stability of anticoagulation for oral vitamin K antagonist treatment. Recently, how intestinal bacteria affect the response of the vitamin K antagonist remains unclear. In this review, we reviewed the research, focusing on the physiology of vitamin K in the anticoagulation treatment, and investigated the potential pathways of intestinal bacteria affecting the reaction of the vitamin K antagonist.

## Introduction

Vitamin K antagonist (VKA) has been found as one of the most extensively used first-line oral anticoagulants (1). Since VKA has a narrow therapeutic range, the international normalized ratio (INR) should be monitored when VKA is used, and the dose should be regulated in time to avoid adverse reactions (e.g., bleeding and embolism). However, VKA still has a high risk of adverse reactions. Statistics suggest that warfarin (the most commonly used VKA) has led to the largest number of emergency department visits for adverse drug events in the United States (2).

The individual dose difference of VKA has been considered the major contributors to the adverse reactions. Existing studies have found that VKA dose requirements are dependent on genetics and clinical factors (3–6). Based on the above factors, researchers have established the pharmacogenomics-guided dosing algorithms for VKA, whereas the clinical value of the above algorithms has been controversial (7–9). Retrospective studies have found that the pharmacogenomics-guided dosing algorithm has a higher VKA stable dose predictive power than the fixed-dose approach and the clinical algorithm (4), while the prospective study results have not yet been satisfying (10–14). The dissatisfactory clinical effect of VKA dosing algorithm primarily arises from the insufficient contribution of the factors included in the algorithm, thus leading to the low prediction accuracy of the algorithm. Therefore, the potential mechanism of VKA dose change should be studied in depth.

Over the past two decades, researchers have primarily identified the possible factors of VKA response at the genetic level, while the effect arising from other levels is ignored. However, except for polymorphisms in *CYP2C9*, *VKORC1*, *CYP4F2*, and *GGCX*, few genetic factors can be included in the dosing algorithm (15). To find more factors and further optimize the VKA dosing algorithm, more attention should be paid to the non-genetic level. As reported by numerous studies, VKA reactions are significantly correlated with gut microbiota. The major effect of VKA is to antagonize vitamin K (VK), which is absorbed by the intestine. Moreover, menaquinone, a vital type of VK for the human body, is mainly synthesized by intestinal bacteria. A combined use of VKA and antibiotics may result in an increase in INR, thus elevating the risk of bleeding (16–20). Accordingly, there may be a potential correlation between intestinal microbiome and VKA response. Through the research on pharmacomicrobiomics of VKA, a new thought may be provided to clarify the mechanism of individual variability of VKA response. In this review, the research focusing on the physiology of VK in the anticoagulation treatment was reviewed, and the potential pathways of intestinal bacteria affecting the reaction of VKA were investigated. Thus, this review is expected to lay a new scientific basis for improving the individualized medication of VKA.

## Methods

In this narrative review, the focus was placed on the available *in vivo* and *in vitro* studies, as well as reviews. Medical subject headings (MeSH), text word, and Boolean calculation search were conducted on the Pubmed database. The retrieval type applied in the database consisted of [(“vitamin K” OR “phylloquinone” OR “menaquinone”) AND (“VKA” OR “warfarin” OR “acenocoumarol” OR “phenprocoumon” OR “INR”)] OR [(“vitamin K” OR “phylloquinone” OR “menaquinone”) AND (“intestin*” OR “gut”) AND (“bacteria” OR “flora” OR “microbiome” OR “microbiota”)] OR [(“VKA” OR “warfarin” OR “acenocoumarol” OR “phenprocoumon” OR “INR”) AND (“intestin*” OR “gut”) AND (“bacteria” OR “flora” OR “microbiome” OR “microbiota”)]. Furthermore, related publications in the reference of the eligible publications and the mechanism research of intestinal bacteria were included as well.

## Types and Sources of Vitamin K

Vitamin K refers to a group of liposoluble vitamin with a wide variety of biology activities, which include playing a certain role in several coagulation factor syntheses, participation in osteocalcin carboxylation, promoting the transition of osteoblasts to osteocytes, limiting osteoclastogenesis and preventing vascular calcifications (21, 22). Naturally occurring VK primarily has two forms, including phylloquinone and menaquinone. All types of VK have a 2-methyl-1,4-naphthoquinone structure, which is termed menadione. Phylloquinone contains a phytyl side chain, consisting of 4 prenyl units, while menaquinone covers an isoprenoid structure side chain with different lengths and degrees of saturation at the 3-position (23). Phylloquinone of humans is largely derived from the diet (24). In accordance with the number of isoprenoid units, menaquinone is termed menaquinone-n, and 12 types of menaquinone-n (MK-4 to MK-15) have been generally reported (25). MK-4 has been found as the most common menaquinone in humans, as well as the only menaquinone converted by phylloquinone (26). The other menaquinone is synthesized by some obligate and facultative anaerobic bacteria (27). Except for MK-4, the other menaquinone in humans is mostly synthesized by intestinal bacteria.

The concentration of VK in plasma and feces has been generally evaluated to indicate the status of vitamin K in humans. In plasma, the concentrations of phylloquinone, MK-4, and MK-7 have been frequently quantified, while the other menaquinone isoforms have been rarely investigated due to the low plasma concentration (28). In feces, almost all types of VK can be quantified, and the concentration level is significantly higher than that in plasma. In humans, there are significant population and individual differences of VK concentration levels. The mean plasma concentrations of phylloquinone have been reported to range from.22 to 8.88 nmol/L. In most studies, the concentrations were lower than 2 nmol/L (29). Diet and medication have a significant effect on the *in vivo* levels of VK.

## Vitamin K and Anticoagulant Therapy

**FIGURE 1:**
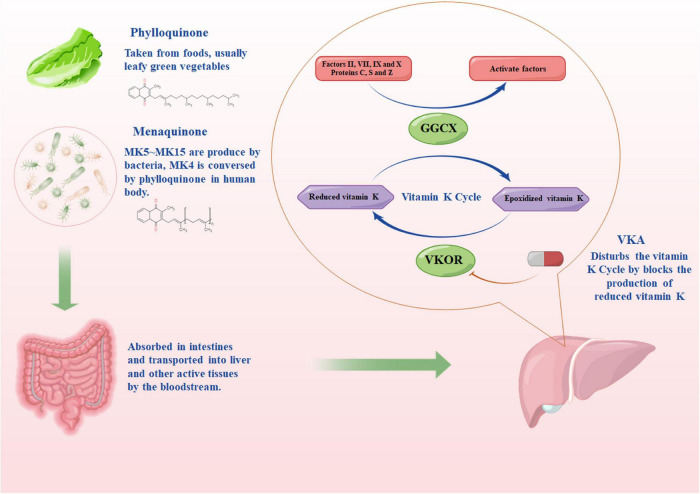
The source of vitamin K and the pharmacodynamic pathway of vitamin K antagonist. MKn, menaquinone, n is the number of isoprenoid units; GGCX, the gamma-glutamyl carboxylase enzyme; VKOR, vitamin K epoxide reductase. The figure was drawing by Microsoft PowerPoint 2016, and the materials were provided by Figdraw (www.figdraw.com).

Vitamin K is of critical significance to VK-dependent clotting factors, which include prohemorrhagic factors (II, VII, IX, and X) and antithrombotic factors (protein C and protein S) that work together to clot the blood (30). VKA is capable of inhibiting the activity of vitamin K epoxide reductase (VKOR) and hindering the production of VKH_2_ (an active form of VK) (31, 32). Under a normal condition of the VK cycle, the gamma-glutamyl carboxylase converts glutamate residues into γ-carboxyglutamate residues through the cofactor VKH_2_, while facilitating the activation of VK-dependent proteins (26). During the carboxylation reaction, VKH_2_ is oxidized into vitamin K 2,3-epoxide (an inactive form of VK). Subsequently, the inactive VK is reduced to VKH_2_ by VKOR to complete the cycle (Figure 1). Impacted by the lower half-life of proteins C and S compared with factors II, VII, IX, and X, the patient is placed in a pro-thrombotic state and then converted into an anticoagulant state when receiving VKA therapy (30).

The change of VK levels has a significant effect on the anticoagulant effect of VKA. A clinical study in Japanese population reported that the plasma MK-4 concentration showed a negative correlation with the warfarin sensitivity index (the ration of INR and the plasma-unbound concentration of each warfarin enantiomer) (33). Another study in European population found that the plasma phylloquinone concentration in the group with a high dose of warfarin was significantly higher than that in the group with a low dose of warfarin (34). In addition, daily VK intake has an effect on the VKA response. Low daily VK intake increases the risk of unstable anticoagulation control in patients treated with VKA, while daily VK supplementation improves anticoagulation control (35–42). Sconce et al. found that, compared with patients with a stable control of anticoagulation, patients with an unstable control had a poor dietary intake of VK (43), and patients receiving 150-μg phylloquinone one time daily had a better anticoagulation control than those patients treated with placebo (38). Menaquinone supplementation has also been considered a way to improve the anticoagulant management for VKA. MK-7 has been the most widely studied menaquinone isoform in clinical practice (39, 44). MK-7 has much more stable serum levels than phylloquinone because of its long elimination half-life (44). Low-dose supplements of MK-7 could also improve the anticoagulant management in patients (39). However, not all patients benefit from VK supplementation, and the dose supplementation of VK improves the stability of VKA therapy in patients with unstable INR only (45).

Besides the dose of VK intake, the stability of VK intake is another vital factor in anticoagulation stability (46). Sudden changes of VK intake may elevate the risk of potential bleeding and thromboembolic complications during VKA therapy (46). Two case reports described that changes in the dietary intake of VK would lead to diffuse bruising or thrombosis, and even result in myocardial infarction (47, 48). Thus, clinicians have generally advised patients who need long-term anticoagulation therapy to control the intake of food rich in VK to ensure the anticoagulant stability of VKA.

## Vitamin K Biosynthesis in the Intestinal Bacteria

There are two ways for intestinal bacteria to synthesize VK, including the Men pathway and the Mqn pathway. In the Men pathway, 2-demethylmenaquinone serves as a precursor for menaquinone synthesis, while the Mqn pathway is completed through futalosine (49–51). Nine types of enzymes (MenA-MenI) are involved in the Men pathway, while the Mqn pathway has five (MqnA-MqnE). In the Human Microbiome Project, 254 genomes of intestinal bacteria have been sequenced. About 24 genomes of *Firmicutes* and *Proteobacteria* have a complete Men pathway (with all MenA-MenI encoded genes), and 10 genomes of *Firmicutes*, *Proteobacteria*, and *Bacteroidetes* have a complete Mqn pathway (52).

In mammals, different intestinal bacteria have genes encoding different enzymes, thus producing different menaquinone isoforms. For instance, *Eubacterium lentum* synthesizes MK-6, *Veillonella* primarily produces MK-7, the *Escherichia coli* mainly produces MK-8, while MK-10 and MK-11 are the major isoforms in *Bacteroides* (53–56). As a result, different compositions of intestinal microbiome produce menaquinone isoforms at different concentrations. According to a recent shotgun metagenomic sequencing study, McCann et al. found that the elderly individuals could be assigned into 4 clusters in accordance with a similar presence and prevalence of menaquinone biosynthesis genes (57). Significant differences in menaquinone isoforms concentration in fecal were found among 4 clusters, except for MK-4, MK-6, and MK-7 (57). It is noteworthy that different menaquinone isoforms may exhibit different biological functions. The total concentrations of menaquinone do not covary with cognition in elderly individuals, whereas certain menaquinone isoforms (e.g., MK-12 and MK-13) are positively correlated with cognition (57).

## Intestinal Bacteria and Anticoagulant Response of the Vitamin K Antagonist

A considerable number of bacteria live in human intestines, especially in the colon (58, 59). Gut microbiome participates in various biological processes in the human body, which cover the development of immune system, the regulation of nervous system, and the biosynthesis of vitamins (vitamins K and B) (60–62). Moreover, the gut microbiome has been confirmed to interact with liver *via* the intestine-liver axis, and it is reported to be correlated with cirrhosis, liver’s antitumor immune function, etc. (63–65). The above results indicated a potential correlation between microbiome and liver function. Since VKA is mainly metabolized and has the VK antagonistic effect in liver, the intestinal bacteria may also have an effect on the anticoagulant response of VKA.

Some studies have reported the indirect correlation between intestinal bacteria and the anticoagulant response of VKA. Numerous antibiotics (e.g., ciprofloxacin, levofloxacin, metronidazole, and fluconazole) have been found to significantly affect the anticoagulant effect of VKA (16, 19, 66, 67). As compared with patients that do not use antibiotics, the patients using antibiotics during warfarin therapy have a significantly higher risk of hemorrhage (16–19). Furthermore, small intestine bacterial overgrowth (SIBO) affects the anticoagulant therapy of warfarin (68). Giuliano et al. reported that patients with SIBO would require a higher warfarin dose compared with patients without SIBO, whereas Emidio et al. achieved the opposite results (34, 69). In human gut, some bacteria are correlated with the synthesis of menaquinone, some bacteria would cause chronic damage of the small bowel mucosa, and some bacteria would impair the absorption of bile acids and VK (69). Accordingly, different research results may be dependent on the types of bacteria abnormal overgrowth in small intestines.

At present, only two studies reported the direct correlation between intestinal bacteria and the anticoagulant response of VKA. In an *in vitro* study, the researchers tested the relationship between 76 intestinal bacteria and 271 drugs, and they found that nearly 2/3 of the 271 drugs could be metabolized by one or more bacteria (70). According to the results, warfarin was found to be metabolized by *Bacteroides vulgatus*, *Collinsella aerofaciens*, *Anaerotruncus colihominis*, *Edwardsiell atarda*, and *Bacteroides fragilis in vitro*. In another study, an association analysis was conducted between intestinal bacteria and warfarin response in patients with heart valve replacement. They found that, compared with the group with a low dose of warfarin, the relative abundance of genus *Escherichia-Shigella* was significantly higher in the group with a high dose of warfarin, while the genus *Enterococcus* was enriched in the low dose group (71). Further analysis indicated that the amount of menaquinone synthesized by intestinal bacteria in the group with a high dose of warfarin was significantly higher than that in the low dose group.

Besides directly affecting the anticoagulant effect of VKA by participating in the coagulation pathway, menaquinone can indirectly affect the efficacy of VKA through pregnane X receptor (PXR). PXR is a nuclear receptor highly expressed in liver and intestines. *in vitro* studies have confirmed that menaquinone could activate PXR (72–75). PXR activation would upregulate the expression of cholesterol transporter Niemann-Pick C1-Like 1 (NPC1L1) in intestinal (76). CD36 refers to a known PXR target gene. Deficiency of PXR decreases CD36 expression in mice, while PXR activation upregulates CD36 expression (77–79). Another *in vitro* study reported that PXR activation could downregulate the expression of SR-BI (80). All of the above three proteins play vital roles in VK absorption in intestines (81, 82). However, since PXR activation has different effects on the above three proteins, how menaquinone affects the absorption of VK *in vivo* should be further investigated. Besides, activation of PXR could upregulate the expression of CYP2C9 in liver (83, 84). CYP2C9 refers to the main metabolic enzyme of VKA, so the concentration of menaquinone in the liver may affect the metabolism of VKA, thus having an influence on the anticoagulant effect.

In general, intestinal bacteria may affect the VKA response by metabolizing the drug in intestinal or regulating the synthesis of menaquinone. Nevertheless, due to the high bioavailability of VKA (nearly 100%), the metabolism of VKA in intestines is negligible (85, 86).

## Intestinal Bacteria Metabolites and Anticoagulant Therapy

Another possible approach to intestinal bacteria affecting the VKA response may be through short-chain fatty acids (SCFAs). SCFAs have been reported as the major type of intestinal bacteria metabolites and derived primarily from intestinal bacteria fermentation of dietary fibers. SCFAs play pivotal roles in many biological processes, consisting of host energy metabolism, cell proliferation, and immune system regulation (87, 88). The main three types of SCFAs produced by intestinal bacteria include butyrate, propionic, and acetic acids, and butyrate has been the most extensively studied SCFAs type.

Intestinal disorders might affect the absorption of VKA and VK and then influence the VKA response. Butyrate serves as a preferred fuel for colonocytes (89). The deficiency of butyrate will lead to nutritional deficiency of colonic epithelial cells and cause colitis (90). Moreover, butyrate protects intestinal mucosa by upregulating the secretion of intestinal mucins and trefoil factors (91). As a result, butyrate plays a vital role in intestinal homeostasis maintaining, which is correlated with the VKA response. Moreover, existing studies found that butyrate could reduce the absorption of dietary cholesterol by downregulating the expression of NPC1L1 in the intestines (92, 93). NPC1L1, a key protein affecting cholesterol uptake, refers to a therapeutic target of dyslipidemia, while ezetimibe serves as a selective inhibitor of NPC1L1. Researchers have found that a combined use of ezetimibe could increase the anticoagulant activity of warfarin (94). Further studies showed that NPC1L1 is a regulatory factor of intestinal phylloquinone absorption (82, 95, 96). *In vitro* experiments found that overexpression of NPC1L1 significantly increased the absorption of phylloquinone in the intestinal cell line (82). In *NPC1L1* gene knockout mice, the concentration of VK is significantly decreased in liver and plasma (82). Moreover, butyrate refers to a well-known Peroxisome Proliferator-Activated Receptor (PPAR) agonist, and activation of PPARα and PPARβ will decrease the expression of NPC1L1 in intestines (92, 97–99). The above results suggest that butyrate may affect the VKA response by regulating VK absorption.

**FIGURE 2:**
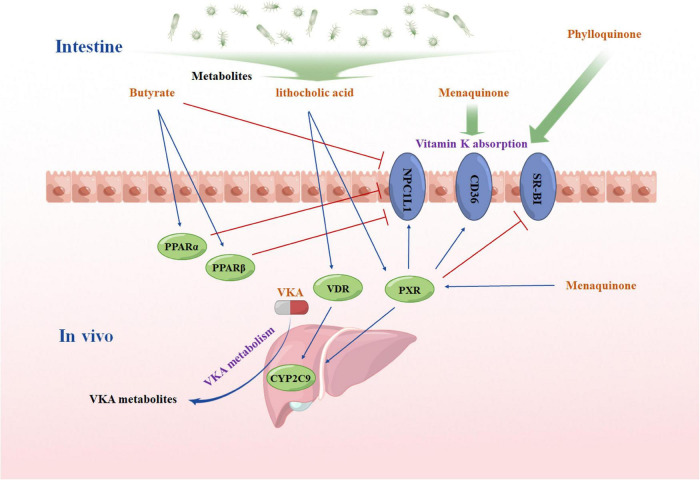
A hypothesis diagram of intestinal microbiome affects the anticoagulant response of vitamin K antagonist VKA, vitamin K inhibitors; PPARα, the peroxisome proliferator-activated receptor α; PPARβ, the peroxisome proliferator-activated receptor β; PXR, pregnane X receptor; VDR, vitamin D receptor; NPC1L1: NPC1like intracellular cholesterol transporter 1; SR-BI: scavenger receptor Class B Member 1; CYP2C9, cytochrome P450 Family 2 Subfamily C Member 9. The figure was drawing by Microsoft PowerPoint 2016, and the materials were provided by Figdraw (www.figdraw.com).

Another metabolite that may affect VKA response is lithocholic acid, which is a secondary bile acid produced by intestinal bacteria (100, 101). Same as menaquinone, lithocholic acid could activate PXR (72). Moreover, lithocholic acid is also vitamin D receptor (VDR) agonists (102, 103). Consistent with PXR, VDR is a drug-activated nuclear receptor, and has been shown to mediate the transcriptional upregulation of CYP2C genes (104). Thus, lithocholic acid can regulate CYP2C9 in liver, and then affect the anticoagulant effect of VKA. Figure 2 shows the possible mechanism of how intestinal microbiome affects the anticoagulant response of VKA.

## Discussion

In this review, the correlations between VKA, VK and intestinal bacteria were investigated. VK has been found as a vital cofactor in the activation of clotting factors. Phylloquinone and menaquinone are found as two major types of VK. The intestinal bacteria may have an effect on the VKA response by producing menaquinone, affecting VK absorption, regulating liver function, etc. Based on the above results, we speculated that there might be significant correlations between VK, intestinal bacteria, and the VKA response. However, the underlying mechanisms still need to be clarified. Whether intestinal bacteria have an effect on the pharmacokinetics and pharmacodynamics of VKA *in vivo* remains unclear, so more pieces of pharmacomicrobiomics research should be conducted.

The current research results revealed that intestinal bacteria could potentially become a warfarin response prediction biomarker. Integration of intestinal bacteria and genetic and clinical factors may improve the accuracy of warfarin dose prediction algorithm and help predict the INR stability of patients after taking warfarin. Intervention of intestinal microbiome may improve the stability of INR.

In this review, the only focus was placed on the correlation between intestinal bacteria and VKA. Impacted by the extensive role of intestinal microbiome, intestinal bacteria may affect the efficacy of other anticoagulants by directly metabolizing drugs, bioaccumulation drugs, or other ways (70, 105). However, the current pharmacomicrobiomics studies of anticoagulants have been rare. To investigate the effect arising from intestinal bacteria on anticoagulant drug response, more research should be conducted in this field. Moreover, although VK status in human body is significantly correlated with VKA response, we can only detect VK concentration in blood and feces for most patients with anticoagulant at present; it is hard to evaluate the systemic VK status and the pleiotropic effects of VK in the body. In the future, novel evaluation methods for global VK status and effects should be developed.

## Author Contributions

XL and HY designed the outline of this manuscript and reviewed and edited the manuscript. YC and HY were the major contributors to the writing of the initial manuscript. HZ, W-HH, and X-HC designed the figures. DL, Y-JL, and Si-Zhao prepared the figures. H-HZ and F-YL selected and organized the references. WZ reviewed and amended the references. All authors contributed to the article and approved the submitted version.

## Conflict of Interest

The authors declare that the research was conducted in the absence of any commercial or financial relationships that could be construed as a potential conflict of interest.

## Publisher’s Note

All claims expressed in this article are solely those of the authors and do not necessarily represent those of their affiliated organizations, or those of the publisher, the editors and the reviewers. Any product that may be evaluated in this article, or claim that may be made by its manufacturer, is not guaranteed or endorsed by the publisher.
